# Leptin Improves Parameters of Brown Adipose Tissue Thermogenesis in Lipodystrophic Mice

**DOI:** 10.3390/nu13082499

**Published:** 2021-07-22

**Authors:** Annett Hoffmann, Thomas Ebert, Mohammed K. Hankir, Gesine Flehmig, Nora Klöting, Beate Jessnitzer, Ulrike Lössner, Michael Stumvoll, Matthias Blüher, Mathias Fasshauer, Anke Tönjes, Konstanze Miehle, Susan Kralisch

**Affiliations:** 1 Medical Department III-Endocrinology, Nephrology, Rheumatology, University of Leipzig Medical Center, 04109 Leipzig, Germany; thomas.ebert@ki.se (T.E.); gesine.flehmig@medizin.uni-leipzig.de (G.F.); beate.jessnitzer@medizin.uni-leipzig.de (B.J.); ulrike.loessner@medizin.uni-leipzig.de (U.L.); michael.stumvoll@medizin.uni-leipzig.de (M.S.); matthias.blueher@medizin.uni-leipzig.de (M.B.); anke.toenjes@medizin.uni-leipzig.de (A.T.); konstanze.miehle@medizin.uni-leipzig.de (K.M.); susan.kralisch@medizin.uni-leipzig.de (S.K.); 2 Department of General, Visceral, Transplant, Vascular and Pediatric Surgery, University Hospital Würzburg, 97080 Würzburg, Germany; hankir_m@ukw.de; 3 Division of Renal Medicine, Department of Clinical Science, Intervention and Technology, Karolinska Institutet, 17177 Solna, Sweden; 4 Helmholtz Institute for Metabolic, Obesity and Vascular Research (HI-MAG) of the Helmholtz Zentrum München at the University of Leipzig, 04109 Leipzig, Germany; nora.kloeting@helmholtz-muenchen.de; 5 Institute of Nutritional Science, Justus-Liebig-University, 35390 Giessen, Germany; mathias.fasshauer@ernaehrung.uni-giessen.de

**Keywords:** lipodystrophy, leptin, brown adipose tissue, thermogenesis, uncoupling protein 1, sympathetic nervous system

## Abstract

Lipodystrophy syndromes (LD) are a heterogeneous group of very rare congenital or acquired disorders characterized by a generalized or partial lack of adipose tissue. They are strongly associated with severe metabolic dysfunction due to ectopic fat accumulation in the liver and other organs and the dysregulation of several key adipokines, including leptin. Treatment with leptin or its analogues is therefore sufficient to reverse some of the metabolic symptoms of LD in patients and in mouse models through distinct mechanisms. Brown adipose tissue (BAT) thermogenesis has emerged as an important regulator of systemic metabolism in rodents and in humans, but it is poorly understood how leptin impacts BAT in LD. Here, we show in transgenic C57Bl/6 mice overexpressing sterol regulatory element-binding protein 1c in adipose tissue (Tg (aP2-nSREBP1c)), an established model of congenital LD, that daily subcutaneous administration of 3 mg/kg leptin for 6 to 8 weeks increases body temperature without affecting food intake or body weight. This is associated with increased protein expression of the thermogenic molecule uncoupling protein 1 (UCP1) and the sympathetic nerve marker tyrosine hydroxylase (TH) in BAT. These findings suggest that leptin treatment in LD stimulates BAT thermogenesis through sympathetic nerves, which might contribute to some of its metabolic benefits by providing a healthy reservoir for excess circulating nutrients.

## 1. Introduction

Lipodystrophy (LD) is an acquired or congenital condition characterized by a partial or complete lack of adipose tissue [1]. Patients with LD suffer from a number of metabolic disorders similar to morbid obesity such as severe insulin resistance, hyperlipidemia, and hepatic steatosis due to the ectopic accumulation of fat in skeletal muscle, pancreas, and liver [1]. In addition, because of the lack of adipose tissue, several metabolically important adipokines are dysregulated in patients with LD, including leptin [2], adiponectin [2], and progranulin [3], amongst others [4].

Because of its status as the archetypal adipokine, the viability of leptin replenishment as a treatment for LD patients was first tested almost twenty years ago [5]. Twice daily subcutaneous administration of recombinant leptin (metreleptin) to 9 patients mainly with acquired or congenital generalized LD for 10 days markedly reduced fasting plasma HbA1c and triglycerides as well as liver volume in association with improved insulin sensitivity and appetite suppression [5]. These findings were subsequently confirmed and extended in a larger, long-term study [6]. Consequently, metreleptin was approved by the FDA for the treatment of LD in 2014 [7] and by the EMA in 2018 [8]. Interestingly, since then, leptin has been shown to correct other abnormalities in LD, such as infertility [9], renal failure [10], and vascular dysfunction [11]. Leptin could therefore exert additional metabolic benefits in LD through targeting various different tissues.

Brown adipose tissue (BAT) is a type of adipose tissue mainly found in the interscapular region in small rodents and in the supraclavicular region in adult humans [12]. Unlike white adipocytes, which store energy in the form of triglycerides, brown adipocytes expend energy due to their high mitochondrial content and the enriched expression of the thermogenic, inner mitochondrial molecule uncoupling protein 1 (UCP1) [13]. Beyond maintaining body temperature, BAT plays an important role in regulating energy, glucose, and lipid homeostasis due to its avid uptake and catabolism of circulating nutrients when activated [14]. The classical stimuli for BAT are cold exposure and feeding through the activation of sympathetic nerve fibers [15]. Chronic leptin treatment also stimulates BAT thermogenesis in leptin-deficient *ob*/*ob* mice by increasing sympathetic innervation and activation [16]. Numerous pharmacological approaches are thus being considered to stimulate BAT thermogenesis for the treatment of metabolic disease by safely mimicking sympathetic nervous system stimulation.

Transgenic mice overexpressing the amino terminal domain of the transcription factor sterol regulatory element-binding protein 1c in adipose tissue (Tg (aP2-nSREBP1c)) are an early model of congenital LD [17]. These mice have a ~65% reduction in epididymal white adipose tissue (WAT) mass, while their BAT becomes markedly enlarged and whitened [17]. Leptin treatment to Tg (aP2-nSREBP1c) mice for 12 days was found to reduce food intake, body weight, plasma insulin, and glucose levels as well as liver fat, but had no effect on BAT *mUcp1* mRNA expression as determined by Northern blot analysis [18]. Similarly, while numerous studies have consistently shown that leptin treatment markedly improves glucose homeostasis and fatty liver in various mouse models of LD [10,18,19,20,21,22,23,24], the effects on energy expenditure have been less positive. Specifically, leptin treatment did not affect energy expenditure in mice with LD from conjugated linoleic acid treatment [21] or deficiency in 1-acylglycerol-3-phosphate O-acyltransferase 2 (AGPAT2) [20] as well as body temperature in mice with LD due to overexpression of the transcriptional repressor A-ZIP/F-1 [25]. These studies, however, were either limited by their relatively short duration of leptin treatment and/or the rather superficial analysis or lack of analysis of BAT. To address these shortcomings, we chronically administered leptin to Tg (aP2-nSREBP1c) mice and analyzed their BAT gene and protein expression of various thermogenic/sympathetic markers.

## 2. Materials and Methods

### 2.1. Animals

The local ethics committee (Regierungspräsidium Leipzig) of the state of Saxony approved the protocol of all animal experiments (37/12 and 27/16). All treatments were performed in the Medical Experimental Center of the University of Leipzig. All mice were on a C57Bl/6 background and were fed ad libitum with a modified, cholesterol-enriched semisynthetic diet (Sniff, Soest, Germany) starting at the age of 4 weeks. They were maintained in a room under pathogen-free conditions with a controlled temperature of 21 ± 1 °C and a 12:12 h light/dark cycle (6 a.m./6 p.m.).

Eight-week-old Tg (aP2-nSREBP1c) male mice on a low-density lipoprotein receptor knockout background were randomized into two groups and treated i.p. with recombinant leptin (3.0 mg/kg body weight (BW); R&D Systems, Wiesbaden-Nordenstadt, Germany) or saline daily for 6 to 8 weeks. At 3.0 mg/kg BW/d, leptin has physiological effects, i.e., this dose is sufficient to normalize BW in male leptin-deficient *ob*/*ob* mice on the same background [26]. Treatment was performed in the morning to mimic the dosing regimen used in LD patients [22]. Non-LD littermates on a low-density lipoprotein receptor knockout background served as controls. Food intake was measured on a weekly basis and is presented as the average over the whole treatment period as g chow/kg mouse/d. At the end of the treatment period, mice were fasted overnight and the last saline or leptin injection was performed 30 min before sacrifice. Cardiac blood was collected in tubes containing EDTA and plasma was separated by centrifugation. Interscapular BAT was removed, weighed, and either immerse-fixed in 4% formalin or was snap frozen.

### 2.2. Temperature Measurements

In mice, rectal temperature measurements were performed with a digital thermometer (Science Products GmbH, Hofheim am Taunus, Germany) simultaneously with i.p. injections of leptin or saline after 6 to 8 weeks of chronic treatment.

### 2.3. Immunoblot Analysis

Western blotting was performed on BAT lysates as previously described [27]. Primary antibodies used were Anti-Tyrosine Hydroxylase (TH) Antibody (AB152, Sigma-Aldrich, St. Louis, MI, USA) at a dilution 1:1000 and Anti-Ucp1 antibody (ab23841, Abcam, Cambridge, UK) at a dilution of 1:1000 and analyzed according to Fischer et al. [28]. Each lane stands for a BAT sample from an individual mouse. We performed two Western blots against UCP1 or TH and had an N ≥ 4 per group in total. For quantification, pixels of each individual lane were counted with the GeneTools 4 software (SynGene, Cambridge, UK) and expressed relative to bActin.

### 2.4. Histological Analysis

Fixed BAT samples were embedded in paraffin and cut into 5 µm thick sections. TH was determined by immunohistochemistry using specific TH antibody (AB152, Sigma-Aldrich, St. Louis, MI, USA) at a 1:1500 dilution. Brown immunoperoxidase staining was developed using a DAB chromogen (Agilent, Waldbronn, Germany) and counterstained with hematoxylin (blue). All slides were photographed using Zeiss Axioskop microscope (Carl Zeiss Microscopy GmbH, Jena, Germany).

### 2.5. Quantitative Real-Time PCR Analysis

*Cell death-inducing DFFA-like effector a* (*mCidea*), *iodothyronine deiodinase 2* (*mDio2*), *Peroxisome proliferator-activated receptor gamma coactivator 1-alpha* (*mPgc1a*), *Neuregulin 4* (*mNrg4*), and *uncoupling protein 1* (*mUcp1*) mRNA synthesis was determined relative to *m36b4* by quantitative real-time RT-PCR in a fluorescent temperature cycler (Roche, Heidelberg, Germany) as described previously [26,29]. Primer sequences used are summarized in Supplementary Material Table S1.

### 2.6. Immunoassays

Murine free triiodothyronine (T3) (DRG Diagnostics, Marburg, Germany) and thyroxine (T4) (DRG Diagnostics, Germany) levels in plasma as well as Norepinephrine (Labor Diagnostika Nord, Nordhorn, Germany) levels in BAT were analyzed by Enzyme-linked Immunosorbent Assay. 

### 2.7. Data Analysis and Statistics

Data sets were analyzed using GraphPad Prism 6 (GraphPad Prism Software, San Diego, CA, USA). Values are presented as mean ± standard error of the mean (SEM). Differences were considered significant at *p* *<* 0.05. To identify significant differences between control group and saline-treated LD or saline-treated LD and leptin-treated LD mice, one-way ANOVA followed by post-hoc Bonferroni–Holm test was used. For non-normally distributed data, values were logarithmically transformed prior to statistical testing.

## 3. Results

### 3.1. Impact of Chronic Leptin Treatment on Body Weight, Food Intake, and BAT Weight in LD Mice

**Figure 1:**
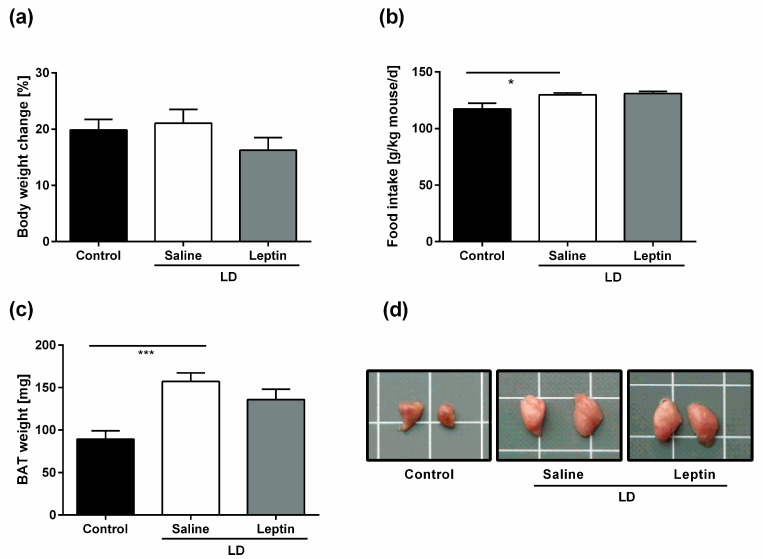
Impact of chronic leptin treatment on body weight, food intake, and BAT weight in LD mice. (**a**) Body weight change (%), (**b**) average food intake over the whole treatment period (g/kg mouse/day), (**c**) BAT weight, and (**d**) representative macroscopic pictures of BAT of control mice, saline-treated LD mice, and 3.0 mg/kg BW/d leptin-treated LD mice over a 6- to 8-week treatment period. Data are presented as mean ± SEM and represent N ≥ 9 per group. White grid: 1 cm^2^. Statistical significance was determined by one-way ANOVA with Bonferroni post hoc test. *: indicates *p* < 0.05; ***: *p* < 0.001.

We first assessed the impact of chronic leptin treatment on body weight and food intake in LD mice. By the end of the 6- to 8-week treatment period, all groups had similar changes in body weight compared to baseline (+19.86 ± 1.9% for control mice, +21.07 ± 2.5% for saline-treated LD mice, and 16.25 ± 2.2% for leptin-treated LD mice; *p* = 0.307) (Figure 1a). Consistent with the hyperphagic state in LD, saline-treated LD mice consumed more food than control mice (129.8 ± 1.6 g/kg mouse/d vs. 117.3 ± 5.2 g/kg mouse/day; *p* = 0.019) (Figure 1b). Leptin treatment in LD mice did not significantly affect food intake at the dose used over the treatment period (3.0 mg/kg BW/d) (131.0 ± 1.8 g/kg mouse/d; *p* > 0.999) (Figure 1b). However, we saw within the first week of treatment a reduction in food intake in the leptin-treated group. This effect diminished from the second week of treatment.

We also assessed the impact of chronic leptin treatment on BAT weight in LD mice. In-line with previous findings [17], the BAT of saline-treated LD mice weighed more than control mice (157.1 ± 10.1 mg vs. 89.3 ± 9.8 mg; *p* < 0.001) (Figure 1c) and phenotypically had a whiter appearance (Figure 1d). Leptin treatment in LD mice did not significantly affect BAT weight (135.9 ± 12.2 mg; *p* = 0.353) (Figure 1c) or its macroscopic appearance (Figure 1d).

### 3.2. Impact of Chronic Leptin Treatment on Body Temperature and BAT UCP1 Expression in LD Mice

**Figure 2:**
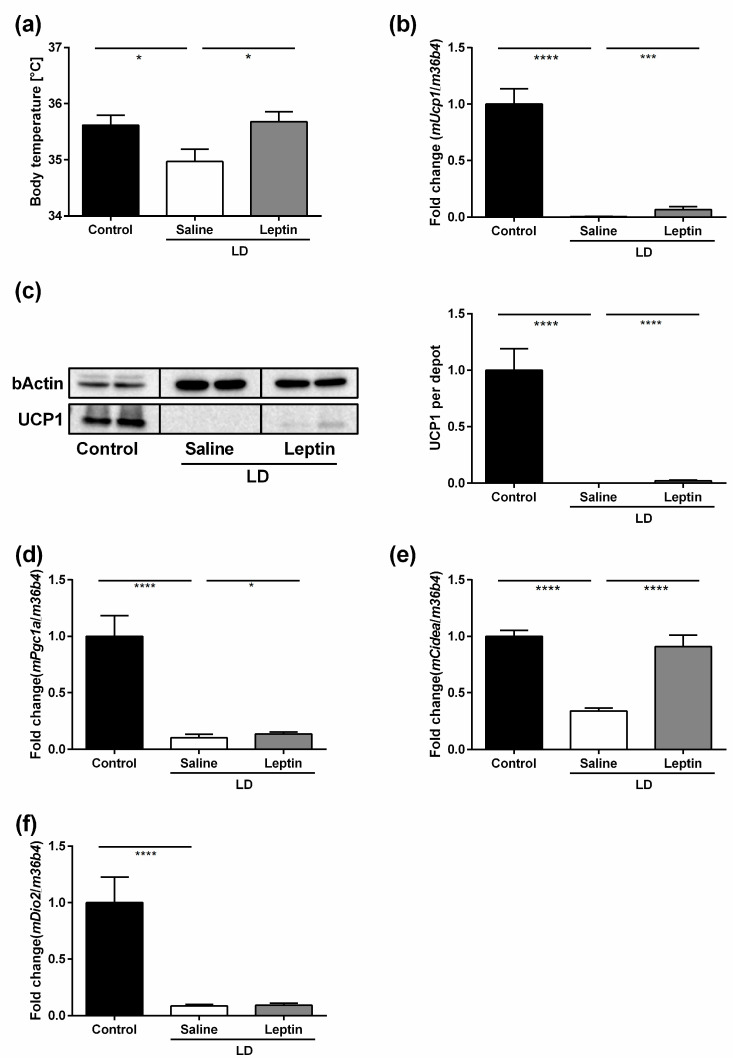
Impact of chronic leptin treatment on body temperature and BAT UCP1 expression in LD mice. (**a**) Body temperature (°C), (**b**) BAT *mUcp1* mRNA expression determined by RT-qPCR, (**c**) UCP1 protein expression determined by Western blot, and (**d**) BAT *mPgc1a*, (**e**) BAT *mCidea*, and (**f**) BAT *mDio2* mRNA expression determined by RT-qPCR of control mice, saline-treated LD mice, and 3.0 mg/kg BW/d leptin-treated LD mice. Data are presented as mean ± SEM and represent N ≥ 4 per group. Statistical significance was determined by one-way ANOVA with Bonferroni post hoc test. *: indicates *p* < 0.05; ***: *p* < 0.001; ****: *p* < 0.0001.

We next assessed the impact of chronic leptin treatment on body temperature and BAT UCP1 expression in LD mice. Saline-treated LD mice had lower body temperature than control mice (34.97 ± 0.22 °C vs. 35.62 ± 0.18 °C; *p* = 0.045) (Figure 2a). Leptin treatment in LD mice raised body temperature (35.68 ± 0.18 °C; *p* = 0.027) (Figure 2a). Consistent with the reduced BAT *mUcp1* mRNA expression previously shown in Tg (SREBP-1c) mice [17], saline-treated LD mice had lower BAT *mUcp1* mRNA expression compared with control mice (*p* < 0.0001) (Figure 2b). Leptin treatment increased BAT *mUcp1* mRNA expression in LD mice (*p* < 0.001) (Figure 2b). A similar pattern for BAT UCP1 protein expression was found between groups (Figure 2c). Further BAT activity markers such as *mPgc1α* (Figure 2d) and *mCidea* (Figure 2e), but not *mDio2* (Figure 2f), were significantly regulated by leptin treatment compared to saline-treated LD mice.

### 3.3. Impact of Chronic Leptin Treatment on BAT Sympathetic Markers in LD Mice

**Figure 3:**
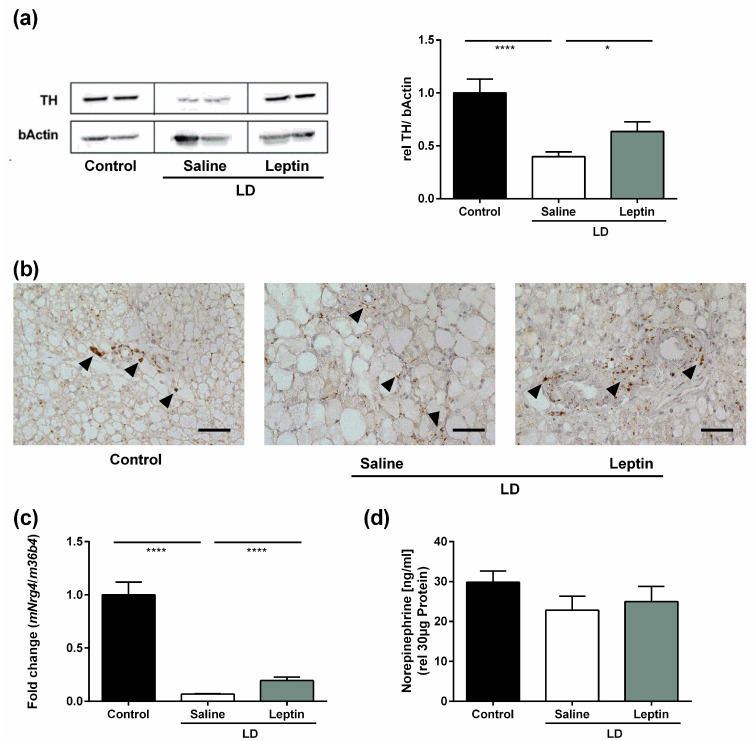
Impact of chronic leptin treatment on BAT sympathetic markers in LD mice. (**a**) BAT TH protein expression determined by Western blot, (**b**) representative immunostaining of TH in BAT, (**c**) BAT *mNrg4* mRNA expression determined by RT-qPCR, and (**d**) BAT norepinephrine levels determined by ELISA of control mice, saline-treated LD mice, and 3.0 mg/kg BW/d leptin-treated mice. Data are presented as mean ± SEM and represent N = 4 per group. Scale bar: 50 µm. Black arrowheads show TH immunoreactivity. Statistical significance was determined by one-way ANOVA with Bonferroni post hoc test. * indicates *p* < 0.05 and **** indicates *p* < 0.0001.

Chronic administration of leptin to *ob*/*ob* mice stimulates BAT thermogenesis by increasing sympathetic innervation and activation [16]. We therefore assessed the impact of chronic leptin treatment on BAT expression of TH, the rate-limiting enzyme in noradrenaline synthesis and a marker of sympathetic nerves, in LD mice. Western blot analysis revealed that saline-treated LD mice had lower BAT TH expression compared with control mice (*p* < 0.0001) (Figure 3a). Leptin treatment in LD mice increased BAT TH expression (*p* = 0.031) (Figure 3a). Immunostaining of TH in BAT confirmed these results (Figure 3b). In line with the increased sympathetic function of leptin-treated mice, brown adipocytes appeared smaller. However, since the overall weight of brown adipose tissue was similar between groups, this is unlikely to be a widespread effect. Additionally, leptin treatment in LD mice increased BAT *mNrg4* expression (Figure 3c), which has been shown to promote BAT thermogenesis through increasing sympathetic innervation [30]. In contrast, ELISA revealed that there was no difference in norepinephrine levels in BAT between groups (29.86 ± 2.8 ng/mL/30 µg protein for control mice vs. 22.80 ± 3.5 ng/mL/30 µg protein for saline-treated LD mice vs. 24.98 ± 3.8 ng/mL/30 µg protein for leptin-treated LD mice; *p* = 0.307) (Figure 3d).

Thyroid hormones also play an essential role in regulating BAT thermogenesis [31]. Additionally, leptin increases circulating thyroid hormones in *ob*/*ob* mice [32]. To determine whether the effects we saw on BAT in LD mice following leptin treatment could be mediated by thyroid hormones, we measured plasma-free T3 and total T4 levels. However, this revealed no significant effect in mice from the different groups (Supplementary Material Figure S1).

## 4. Discussion

In the present study, we found that leptin-treated LD mice had higher body temperature than their saline-treated counterparts. This was associated with increased molecular markers of thermogenesis and sympathetic nerves in BAT.

Optimal BAT thermogenesis requires UCP1 along with the coordinated expansion/activation of sympathetic nerves [13]. It has previously been shown that chronic leptin treatment in *ob*/*ob* mice stimulates BAT thermogenesis by increasing sympathetic innervation and activation through an intricate hypothalamic neuronal network [16]. Our findings raise the possibility that a similar central pathway could be recruited by chronic leptin treatment in LD. It has also been shown that sympathetic innervation of BAT is increased by NRG4 released from brown adipocytes downstream of BMP8b signaling [30]. In line with these findings, we found increased *mNrg4* mRNA expression in BAT of leptin-treated LD mice in association with increased sympathetic nerve and thermogenic markers in BAT. It would be interesting to determine in future studies if leptin cell autonomously regulates *mNrg4* expression in brown adipocytes. Importantly, the distinction between increased sympathetic nerve activation and innervation by leptin was not made in this study. This can be addressed in future studies by performing fluorescence microscopy on delipidated, TH-stained BAT samples [16]. Additionally, the requirement of BAT for the thermogenic effect of leptin in LD mice can be determined by sympathetic denervation with 6-hydroxydopamine or pharmacological blockade of beta-adrenergic receptors with propranolol.

An alternative explanation for the thermogenic effect of leptin in *ob*/*ob* mice is that it prevents heat loss through the tail [28]. Whether this is similarly the case in LD mice is unclear, and can be determined in future studies using thermal imaging.

Previous studies in LD mice showed no effects of leptin treatment on energy expenditure [20,21], body temperature [25], or on BAT *mUcp1* mRNA expression [18]. The difference with the present study could be due to a number of methodological factors such as LD mouse model (AGPAT^−/−^ mice in [20], conjugated linoleic acid-treated mice in [21], and Tg (A-ZIP/F-1) mice in [25]), the duration of leptin treatment (3-week infusion in [20], single injection in [21], and 1-week infusion in [25]), and/or the method to determine thermogenesis (48 to 80 h energy expenditure measurements in [21,25] and core body temperature measurements by telemetry following torpor induced by an overnight fast in [25]). The study of Shimomura et al. [18] is the most similar to the present study since the authors also used Tg (aP2-nSREBP1c) mice. However, leptin was administered for 12 days (vs. 6 to 8 weeks in our study) before BAT *mUcp1* mRNA was measured by Northern blot analysis (vs. RT-qPCR). Our findings thus suggest that while the positive impact of leptin on thermogenesis in LD mice is delayed, it is still of potential clinical relevance since LD patients tend to receive metreleptin treatment for many years.

A recent study in 17 patients with generalized or partial LD showed that twice daily injection of 5 mg metreleptin for 2 weeks actually reduced energy expenditure [33]. Additionally, core body temperature and skin temperature were unaffected by leptin treatment. In contrast, we found in preliminary data a significant increase in body temperature in our patients with partial LD after acute leptin administration and no difference in energy expenditure (data not shown). Future studies using positron emission tomography imaging are therefore warranted to more conclusively determine if BAT function is enhanced by leptin treatment in patients with LD [34].

An important question raised by our findings is what beneficial impact (metabolic or other) increased BAT thermogenesis could have in LD. A major defining characteristic of LD is the ectopic accumulation of fat in liver and other organs due to the lack of adipose tissue. Since BAT is a major sink for circulating nutrients, including lipids, when activated by cold [35,36,37,38], the anti-hyperlipidemia effects of leptin in LD might be partly attributable to increased BAT thermogenesis. Another question relates to the possible heat-insulating effect of adipose tissue. If this were the case, any heat loss in LD through reduced adipose tissue could be counteracted by increased BAT thermogenesis from leptin treatment. However, this is unlikely, as adipose tissue does not appear to have a major insulating role in mice or in humans [39]. Additionally, LD mice have almost no markers of thermogenesis in BAT, arguing against any form of compensatory BAT thermogenesis in the first place. Another set of experiments involving measurement of VO2 consumption and VCO2 production to assess energy expenditure would be desirable in addition to continuous body temperature measurements for 24 h.

In summary, we have presented evidence that chronic leptin treatment stimulates BAT thermogenesis in association with increased sympathetic markers in BAT in LD mice. Future studies can reveal whether this specific effect contributes to some of the metabolic benefits of leptin treatment.

## Data Availability

The data in this manuscript are available on request from the corresponding authors.

## Supplementary Materials

The following are available online at https://www.mdpi.com/article/10.3390/nu13082499/s1, Figure S1: title, Table S1: Primer sequences 5′-3′ used for quantitative real-time RT-PCR analysis; Figure S1. Effects of chronic leptin treatment on circulating thyroid homones in LD mice. Circulating (a) free triiodothyronine (fT3) and (b) thyroxine (T4) determined by ELISA of control mice, saline-treated LD mice, and 3.0 mg/kg BW/day leptin-treated mice. Data are presented as mean ± SEM and represent *N* = 8 per group.
