# Supplementary material for: Leptin Improves Parameters of Brown Adipose Tissue Thermogenesis in Lipodystrophic Mice
Source: Nutrients. 2021 Jul 22;13(8):2499. doi: 10.3390/nu13082499 (PMC8399124; doi:10.3390/nu13082499)
Supplement: Supplementary file 1 [file nutrients-13-02499-s001.zip › nutrients-1285224-supplementary.pdf]

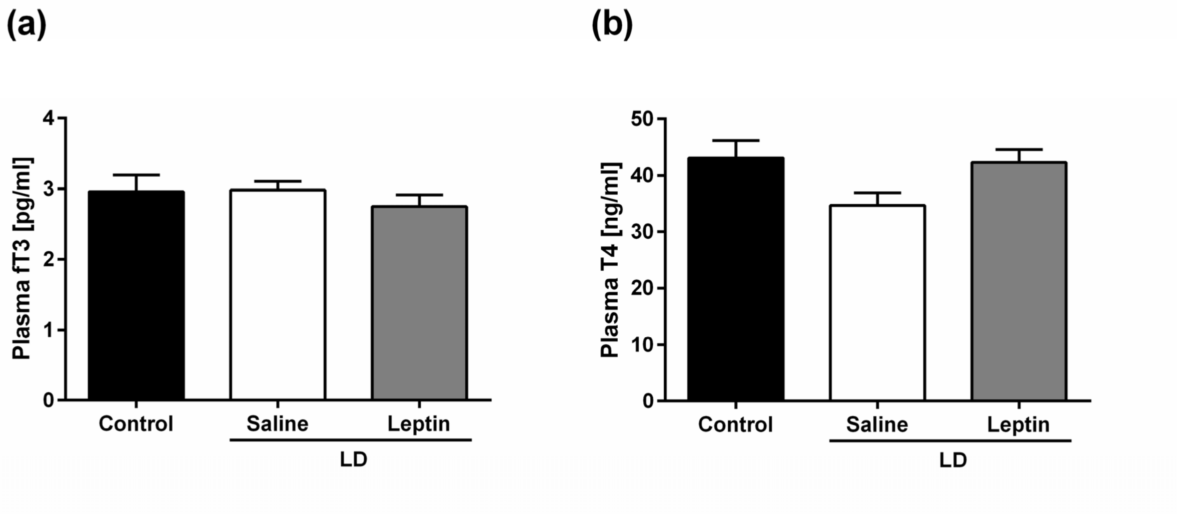

Supplementary Figure S1. Effects of chronic leptin treatment on circulating thyroid homones in LD mice. Circulating **(a)** free triiodothyronine (fT3) and **(b)** thyroxine (T4) determined by ELISA of control mice, saline-treated LD mice, and 3.0 mg/kg BW/day leptin-treated mice. Data are presented as mean ± SEM and represent N = 8 per group.

Gene	Forward sequence	Reverse sequence
36b4	AAGCGCGTCCTGGCATTGTCT	CCGCAGGGGCAGCAGTGGT
Cidea	GCCGTGTTAAGGAATCTGCTG	TGCTCTTCTGTATCGCCCAGT
Dio2	CAGTGTGGTGCACGTCTCCAATC	TGAACCAAAGTTGACCACCAG
Pgc1a	CTTTTGTGGACGGAAGCAAT	GAGTCTTGGGAAAGGACACG
Nrg4	CGACGAGAGAAGCCCATCAT	TCCAGGCCAGTGATGACAGTA
Ucp1	TCTGCATGGGATCAAACCCC	ACAGTAAATGGCAGGGGACG

Supplemental Table S1. Primer sequences 5'-3' used for quantitative real-time RT-PCR analysis.
